# 5FU-loaded PCL/Chitosan/Fe_3_O_4_ Core-Shell Nanofibers Structure: An Approach to Multi-Mode Anticancer System

**DOI:** 10.34172/apb.2022.060

**Published:** 2021-09-29

**Authors:** Mehdi Hadjianfar, Dariush Semnani, Jaleh Varshosaz, Sajad Mohammadi, Sayed Pedram Rezazadeh Tehrani

**Affiliations:** ^1^Department of Textile Engineering, Isfahan University of Technology, Isfahan, Iran.; ^2^Department of Pharmaceutics School of Pharmacy and Pharmaceutical Sciences, Isfahan University of Medical Sciences, Isfahan, Iran.; ^3^Kia Nano Vista Laboratory, Tehran, Iran.

**Keywords:** Nanofibers, Drug delivery systems, Artificial intelligence, Fluorouracil, Chitosan, Magnetic field therapy

## Abstract

***Purpose:*** 5-Fluorouracil (5FU) and Fe_3_O_4_ nanoparticles were encapsulated in core-shell polycaprolactone (PCL)/chitosan (CS) nanofibers as a multi-mode anticancer system to study drug release sustainability. The structure of the core-shell drug delivery system was also optimized according to drug release behavior by artificial intelligence.

***Methods:*** The core-shell nanofibers were electrospun by a coaxial syringe. Artificial neural network (ANN) was used for function approximation to estimate release parameters. A genetic algorithm was then used for optimizing the structure. Chemical assay of the optimized sample was performed by Fourier transform infrared spectroscopy (FTIR), X-ray diffraction (XRD), and energy-dispersive X-ray spectroscopy (EDX). vibration sample magnetometer (VSM) test was conducted to measure the real amount of loaded magnetic nanoparticles. HepG2 cell cytotoxicity was studied and the results for the optimized samples with and without Fe_3_O_4_ after 72 hours were reported.

***Results:*** Feeding ratio of sheath to core and the amount of CS, Fe_3_O_4_, and 5FU had a statistical effect on nanofibers diameters, which were 300-450 nm. The drug loading efficiency of these nanofibers was 65-86%. ANN estimated the release parameters with an error of 10%. The temperature increased about 5.6°C in the alternative magnetic field (AMF) of 216 kA.m^-1^~300 kHz and 4.8°C in the AMF of 154 kA.m^-1^~400 kHz after 20 minutes. HepG2 cell cytotoxicity for the optimized samples with and without Fe_3_O_4_ after 72 hours were 39.7% and 38.8%, respectively.

***Conclusion:*** Since this core-shell drug release system was more sustainable compared to the blend structure despite the low half-life of 5FU, it is suggested to utilize it as post-surgical implants for various cancer treatments such as liver or colorectal cancer in the future. This system is capable of providing chemotherapy and hyperthermia simultaneously.

## Introduction

Despite current cancer biological treatments progress, yet it takes millions of lives around the world every year.^1^ Targeted drug delivery systems can release pharmaceutical agents on the desired spot which can reduce the systemic effect. Moreover, controlled delivery of drug can increase concentration of drug around the tumor while no degradation of pharmaceutics occurs in intact tissues.^2^ Hyperthermia can be considered as a supplementary treatment in addition to common therapies such as chemotherapy and radiotherapy. In addition, it increases the sensitivity of the cancer cells to anticancer drugs which leads to a decrease in the size of tumor according to related studies.^3^

Polymeric nanofibers have gained great attention for various biomedical applications.^4^ These structures are capable of being utilized in drug releasing systems which can be encapsulated by different therapeutic agents such as chemotherapy drugs.^5^ Applying electrospun nanofibers, as anticancer drug carriers, has advantages such as increasing drug concentration around tumor with less amount of drug, prolonging the effect of drug on the target area, and decreasing toxicity on the non-tumorous organs.^6^ Other benefits include; more efficient drug loading in comparison with other drug carriers, broad special surface offering a readier release for hydrophobic drugs, flexible geometry, low cost, biodegradability, and high water absorption.^1,6^

Nanofibers loaded by anti-cancer drugs for different cancer treatments have been utilized in a number of studies. A wide range of biodegradable and biocompatible polymers have been applied for such drug delivery systems. Polylactic acid (PLA)/polyethylene glycol nanofibers including doxorubicin (DOX)^7^ and hydroxycamptothecin (HCPT)^8^ were studied for cancerous cells treatment. The results showed an increased effect of drug loaded nanofibers in comparison with pure drug. Also nanofibrous structures of chitosan (CS)/polycaprolactone (PCL) including 5FU^1^ or cisplatin^6^ were designed for anti-cancer drug delivery systems. In another work, polyethylene oxide (PEO)/PLA including cisplatin was used for cervical cancer. The results showed a significant decrease in drug concentration in blood circulation system and other organs in contrast with targeted tissue which led to less side effects of the drugs.^9,10^ Core-shell gelatin/polyvinyl alcohol (PVA) nanofibers carrying DOX were used for breast cancer treatment. The results showed lower concentration of drug around heart and kidney after implantation of nanofibers compared to direct injection of the drug which caused less side effects.^11^

A triggered drug release system from nanofibers made of n-isopropyl acrylamide/n-hydroxymethyl acrylamide copolymer having DOX and Fe_3_O_4_ nanoparticles was characterized for skin cancer apoptosis. Thermo-responsive structure of the nanofibers was sensitive to temperature increase and the drug was released in an alternative magnetic field (AMF) after thermal response of Fe_3_O_4_ nanoparticles.^12^ Having considered AMF as a heat source, Fe_3_O_4_ nanoparticles were encapsulated in PCL/CS nanofibers merely for hyperthermia as a supplementary treatment.^13^ Nonetheless, one of the major challenges of using Fe_3_O_4_ nanoparticles for hyperthermia is locating them on the targeted area.

5-Fluorouracil (5FU), which is one of the most widely used antimetabolite chemotherapeutic agents in recent decades, has been employed as an antineoplastic agent in the treatment of several cancers, such as colorectal, breast, head and neck, pancreas and stomach cancers.^14^ Although 5FU is among the superior chemotherapeutic agents for various cancers, there are several disadvantages, such as rapid metabolism, short half-life, low bioavailability, high cell toxicity and inadequate selectivity for cancerous cells, all of which limit the effectiveness of 5FU in cancer chemotherapy.^14^ It is crucial to develop drug delivery systems for 5FU to achieve a better therapeutic effect with fewer side effects and a good targeting effect to overcome the disadvantages of 5FU like its short half-life and poor efficacy.^15^

5FU can enter cells via the same mechanism of facilitated transport as uracil.^16^ Then, 5FU is converted to fluorodeoxyuridine monophosphate (FDUMP), fluorodeoxyuridine triphosphate (FDUTP) and fluorouridine triphosphate (FUTP), which are the active metabolites of 5FU. RNA synthesis and the operation of thymidylate synthase are interrupted by these metabolites. By this action, 5FU can fight cancerous cells.^14,17^ Nonetheless, most of the provided 5FU is catabolized by dihydropyrimidine dehydrogenase (DPD) to dihydrofluorouracil, which is an inactive metabolite and a retarding enzyme for 5FU catabolism and mainly found in liver and cancer cells.^14,18^ Since upregulation of DPD gene expression in cancer cells is associated with 5FU resistance, higher doses of 5FU are needed in cancer cells with acquired drug resistance.^18,19^

A polyacrylic acid (PAA) grafted-chitosan (CS-g)/polyurethane (PU) core-shell nanofiber was loaded with magnetic nanoparticles in shell and temozolomide (TMZ) and paclitaxel (PTX) in core. The nanofibers were then characterized for controlled release system against glioblastoma cancer cells. These nanofibers could provide a combination of chemotherapy and hyperthermia methods for glioblastoma cancer treatment. The cell cytotoxicity indicated that 31.3 and 49.6% of apoptosis cell was occurred for U-87 MG glioblastoma cells treated with CS-g-PAA–TMZ-PTX/PU/magnetic MIL-53 in the absence and presence of AMF, respectively.^20^

The aim of this study was to encapsulate 5FU and Fe_3_O_4_ nanoparticles in core-shell PCL/CS nanofibers as a multi-mode anticancer system which could provide chemotherapy and hyperthermia simultaneously. Despite 5FU has a low half-life of 8 to 20 minutes,^21^ core-shell structure of the nanofiber contributes to a more sustainable release system of the drug in comparison with blend structure, which was studied in previous work.^1^ PCL/CS nanofibers can increase the presence time and concentration of it in the release area. Moreover, Fe_3_O_4_ nanoparticles in nanofibers provide the possibility of hyperthermia in the area where fibers present. Fe_3_O_4_ nanoparticles generate heat more evenly under AMF which can overcome hyperthermia challenges. Therefore, these nanofibers can be utilized as post-surgical implants for various cancer treatments such as liver or colorectal cancer in future.

## Materials and Methods

### Materials

PCL with molecular weight of 70-80 kDa, CS with medium molecular weight of 190-310 kDa, 12 kDa dialysis tube cut-off, and MTT tetrazolium were all purchased from Sigma, the US. 5FU, 98% formic acid, and 99% acetic acid were prepared by Merck, Germany. 20 nm Fe_3_O_4_ nanoparticles coated by acetic acid were obtained from Nanosany Corporation, Iran. Phosphate-buffered saline (PBS) solution was supplied from Cyto Matin Gene (CMG), Iran. Hepatoma cell line (HepG2) was obtained from Pastor Institute, Iran.

### Core-shell nanofiber electrospinning

Core and sheath solutions were prepared separately. Pure 12%wt. PCL with 1, 3, and 5% 5FU concentrations were dissolved in 70:30 formic acid/acetic acid and stirred for 15 minutes for core solution. In order for preparing sheath solution, pure 12%wt. PCL with 2%wt. CS concentrations in different polymer ratios were dissolved in 70:30 formic acid/acetic acid and stirred for 15 minutes. After adding 5FU and stirring for another 15 minutes, various amounts of Fe_3_O_4_ nanoparticles were applied to the solution and mixed mechanically for 5 minutes for obtaining even dispersion. At the end, the solution was placed in an ultrasonic bath (PS-10A Jenken ultrasonic cleaner bath, China) for an additional 15 minutes. The design of experiments (DOE) was conducted by Taguchi method using Minitab. The variables were PCL:CS ratio, nanoparticle and drug percentage, feeding ratio of sheath to core, and magnetic field frequency. Table 1 shows the DOE for drug release parameters study.

**Table 1:** Design of experiment of drug release system

**Experiment code**	**Feeding ratio of sheath to core**	**Polymer ratio in sheath (PCL:CS, v:v)**	**Nanoparticle %**	**Drug %**	**Magnetic field frequency, kHz**
C1	1.25	2:1	1	3	0
C2	1.25	2:1	3	1	400
C3	1.25	2:1	7	5	300
C4	1.25	1:1	1	5	400
C5	1.25	1:1	3	3	300
C6	1.25	1:1	7	1	0
C7	1.25	1:2	1	1	300
C8	1.25	1:2	3	5	0
C9	1.25	1:2	7	3	400
C10	2	2:1	1	1	400
C11	2	2:1	3	5	300
C12	2	2:1	7	3	0
C13	2	1:1	1	5	0
C14	2	1:1	3	3	400
C15	2	1:1	7	1	300
C16	2	1:2	1	3	300
C17	2	1:2	3	1	0
C18	2	1:2	7	5	400

Nanofibers were electrospun through a coaxial syringe with a core needle gauge of 22 and sheath needle gauge of 16. The distance from the needle tip to a 200-rpm rotating drum was 14 cm in a 15 kV applied voltage. The syringe pump and power supply were provided by Pars Nanoris, Iran. The feeding ratio of sheath was 0.08-0.1 mL/h while for core, it was considered according to sheath feeding ratio and DOE. All nanofibers were also produced without drug as for blind samples.

### Characterization of nanofibers

#### Morphology

To investigate nanofibers morphology, scanning electronic microscopy (SEM), (Philips XL30, the Netherlands) and transmittance electron microscopy (TEM), (Philips EM 208S, the Netherlands) images were analyzed. In order to measure fibers diameter, Digimizer software was applied in which 100 fibers from each SEM image were opted randomly.

#### Viscosity and conductivity of sheath solution

10 ml of sheath solution was added to the cup of a Brookfield LVDV-II+Pro Rotational Viscometer, Canada in 25°C with a cone spindle speed of 3rpm in order to measure the viscosity. The conductivity was measured by a Jenway 4510 Bench conductivity meter, China.

#### Hydrophilicity assessment

A 1 µL droplet of water was dripped on the surface of a 1×1 cm^2^ optimized nanofibrous layer sample in a Jikan CAG-10 contact angle goniometer, Iran. A photograph was then taken from the droplet on the surface after 20 seconds. This procedure was repeated 3 times and the contact angle was measured by Digimizer software.

#### Chemical assay

Fourier transform infrared spectroscopy (FTIR) was performed in a Bomem-MB100 spectrophotometer, Canada over the range of 400 to 4000 cm^-1^ after calibrating the apparatus with a potassium bromide (KBr) compressed film to assay chemical structure of optimized nanofibers. Grazing X-ray diffraction (XRD) analysis was conducted by a PANalytical X-ray diffractometer, the Netherlands at a voltage of 40kV with Ni-filtered Cu-Kα radiation from 2θ 10° to 80° to visualize iron oxide structure. Differential scanning calorimetry (DSC) was applied for the optimized nanofibrous layer by a BÄHR Thermoanalyse DSC 302, Germany to assess the capability of the layers for hyperthermia. For DSC, 5 mg of nanofibrous layer with and without 5FU was placed in the sample holder and the temperature was increased with a gradient of 10°C.min^-1^ from 25 to 400°C. Energy-dispersive X-ray spectroscopy (EDX) was done by an EDAX EDS Silicon Drift 2017, the US to demonstrate elemental analysis of the optimized sample.

#### Tensile properties

A tensile tester of Zwick 1446-60, Germany was used for measuring tensile strength, strain, and modulus of the nanofibrous layers. Three samples for each nanofibrous layer were cut into 30×5 mm^2^ and placed between the grippers on a paper frame. The gauge length and test speed were 20mm and 10 mm/min, respectively. The thickness of each specimen was measured for 10 times using a digital micrometer (Insize non-rotating spindle digital micrometer 3631-25, China).

#### Hyperthermia assessment

Magnetic properties of the optimized sample were measured by a vibration sample magnetometer (VSM, Meghnatis-Daghigh-Kavir, Iran). The applied magnetic fields ranged in an interval of ±15kOe at 300°K. In order to investigate nanofibrous layers capability for hyperthermia, thermal behavior of the optimized sample was studied under two AMFs of 154 kA.m^-1^, 400 kHz and 216kA.m^-1^, 300 kHz. For this, 200 mg of the optimized sample was soaked in 2 mL PBS for 30 minutes and then exposed to the AMFs for a 20-minute period.

#### In vitro degradation assessment

Degradation of the optimized sample was assessed according to ASTM F1635-04A. Three samples all with a dimension of 4×4 cm^2^ were prepared and soaked in PBS solution with a pH of 7.4 at 37°C for 24, 48, 96, 240, 504, and 1000 hours. The samples were dried in a sealed and vacuumed desiccator containing silica gel for 24 hours after each time period and then weighed.

### Drug release behavior

#### Loading efficiency

Since proportion of drug was the same in core and sheath, loading efficiency could be considered as for blend nanofibers and was determined according to the previous work.^1^ The Beer-Lambert’s law was verified for the solvent solution (formic acid/acetic acid 70:30 v/v) and the maximum absorption wavelength for 5FU was 266 nm using a UV-mini-1240, Shimadzu, Japan spectrophotometer.

#### In vitro drug release

5FU loaded nanofibers was immersed in PBS with a pH of 7.4 and the calibration curve was verified with Beer-Lambert’s law in PBS at λ_max_ = 266 nm. The samples were placed in dialysis tubes with a cut-off of 12 kDa which were prepared in advanced. Three samples with dimensions of 4×4 cm^2^ for each composition were placed in wrapped tubes containing 10 mL PBS. The tubes were then immersed in beakers containing 30 mL PBS and the whole systems were sealed and placed in a shaking incubator with a rotation speed of 110 rpm in 37°C. The release profiles were investigated within a week after exposing the beakers for 10 minutes to various AMFs according to Table 1 in order to study the effect of AMF on release behavior. For each specimen a relative control sample was prepared without drug. All the above-mentioned procedure was repeated for the optimized sample in pH of 7.4 and 4.4.

#### Release kinetics

The release behavior of nanofibers was studied through zero order, first order, Hixson-Crowell, Higuchi, Korsmeyer-Peppas, and Weibull models.

### Modelling and optimization

#### Theoretical basis

Artificial neural network (ANN) is a more efficient and suitable method in comparison with standard modelling methods such as response surface methodology.^22^ On the other hand, since there was not a precise perception about the way how structural parameters (feeding ratio of sheath to core, polymer ratio in sheath (PCL: CS), nanoparticle and drug percentage) were related to release parameters (burst release [BR], drug loading efficiency, maximum release time and its relevant release amount), ANN was used for function approximation in the definite intervals of structural parameters. A multilayer perceptron ANN was applied since it is a common method in nonlinear regression problems. The number of hidden layers and their neurons could be determined through trial and error.^23^ However, based on Kolmogorov’s theorem, an ANN with one hidden layer was applied.^24^

In order to prevent over fitting, sufficient number of training data are essential. Since training data were limited due to experimental restrictions, Gaussian noise exertion on real data^25^ and k-fold cross validation method^26^ were applied. Finally, Gaussian noise method was selected according to the training error. Another way to prevent overfitting is adopting suitable ANN training method. Early stopping and Bayesian regularization (BR) are the most commonly used methods for ANN training.^27^ In comparison with early stopping method, BR needs no validation data ^23^ and has higher coefficient of determination (R^2^).^27^ Therefore, BR was applied for ANN training. The obtained ANN with the above-mentioned architecture was used for deriving the genetic algorithm fitness function.

Optimizing is defined as finding the best answer regarding the requirements and restrictions of the problems which can be solved through analytical and numerical methods. Classical mathematical methods have some disadvantages as opposed to smart methods such as genetic algorithm. The drawbacks include considering local optimized points instead of the real ones and the necessity for initial answer guess which affects the final results. On the other hand, genetic algorithm is inspired by creatures evolution in which optimizing problems can be solved sufficiently based on the ability to compete, survive, and reproduce.^28^

In genetic algorithm each individual or chromosome is a proposed solution for a problem, and a set of chromosomes is called a population or generation.^29^ The whole data pool is searched to select initial random population, and eventually by crossover and mutation the algorithm will evade to be trapped in local optimized points.^28^ The next generation is then created from the present generation based on fitness value which must be better or, in other words more optimized. This process will be stopped under certain conditions which are defined in genetic algorithm structure parameters.^30^

#### Practical basis

A perceptron ANN with one hidden layer was applied for estimating and approximating release parameters according to structural parameters. The number of input and output neurons was the same as the number of input (structural) and output (release) parameters, respectively. For determining the number of hidden layer neurons, various ANNs with 2-7 neurons in hidden layer were designed. Each ANN was run 10 times and the average of the mean square error (MSE), and R^2^ were reported for training and testing data. The ANN with the least MSE and R^2^ for testing data was selected as the best ANN for modelling whose outputs were used in genetic algorithm for optimizing the structure of core-shell nanofibers. There were constraints on defining the chromosome of the algorithm regarding production restrictions. Chromosomes include feeding ratio of sheath to core with a constraint of [1-2], CS percent in sheath with a constraint of [7-23], nanoparticle percentage with a constraint of [1-7], and drug percentage with a constraint of [1-5].

Fitness function was defined in a way that drug loading efficiency, maximum release time and its relevant release amount were maximum and BR was minimum. Ideally, when fitness value is zero the optimized structure is obtained. Equation 1 shows the fitness function.


(1) $$Fitness\;value=\frac{\left(\frac{\left|100-R_{TR\max}\right|}{100}+\frac{\left|BR\right|}{100}+\frac{\left|TR_{\max}-240\right|}{240}+\frac{\left|100-L_{e}\right|}{100}\right)}{5}$$

Wherein R_TRmax_ was maximum release amount in maximum release time, BR was burst release which was the release amount in the first 30 minutes, TR_max_ was the maximum release time, and L_e_ was drug loading efficiency.

A MATLAB code was used for genetic algorithm whose parameters are population size (20), the number of generations (100), creation function (Roulette wheel), the number of elites (1), cross over fraction (0.8), migration fraction (0.2), mutation fraction (0.2), migration direction (forward), fitness limit (0), function tolerance (10^-6^), and maximum stall generation (5). Stopping conditions consisted of either reaching to 100 generations, or achieving zero for fitness value, or having the best fitness value difference less than the function tolerance for 5 consecutive generations. The algorithm was run 20 times and the best values for gens were reported as the optimized structure.

A real sample was produced based on the result of the optimization process and the release parameters of the practical and theoretical samples were compared in order to evaluate model accuracy.

### Cell assessment

#### Cell culture media preparation

HepG2, a perpetual cell line consisting human liver carcinoma cells, was derived from the liver tissue of a 15-year-old Caucasian male patient who had a well-differentiated hepatocellular carcinoma (HCC). HepG2 cells were cultured in Dulbecco’s modiﬁed Eagle’s medium (DMEM) supplemented with 10% PBS and 1% penicillin-streptomycin at 37°C in an incubator with 5% CO_2_ and 90% humidity. The culture medium was then replaced every two days.^31^ After reaching 80% conﬂuence, the cells culture medium was aspirated from the culture flask. 10 ml of PBS was then lightly added to the flask and aspirated again. Next, 0.25% Trypsin-EDTA was added to the flask and incubated at 37°C for 3 minutes. Finally, in order to deactivate Trypsin-EDTA, 10 mL DMEM containing 10% fetal bovine serum (FBS) was added. Eventually the cells were detached and pipetted into a sterile 15 mL centrifuge tube. Prepared cell culture media were spun at 130 g for 5 minutes. The cells were then counted after aspirating the supernatant and adding DMEM containing 10% FBS to the pellet.^32^

#### Cytotoxicity assay

Cytotoxicity was conducted according to ISO 10993-5. 2×10^4^ cells were seeded in a 96-well plate within 100 µL culture medium for 24 hours. The wells were divided into 4 groups of negative control, positive control, extraction of optimized nanofibers without Fe_3_O_4_, and extraction of optimized nanofibers with Fe_3_O_4_. The cells in positive control group were exposed to 20, 60, 100, 175, and 250 µg/ml of 5FU after aspirating initial culture medium. In the third and fourth groups, nanofibers without and with Fe_3_O_4_ were extracted separately after 24, 48, and 72 hours and the extracted drug was added to the seeded cells after the initial culture medium was pipetted out. Having sterilized by UV, extraction was done by adding 1 mL free-FBS DMEM to a 2×3 cm^2^ nanofibrous mat with an approximate thickness of 10 µm, in which the concentration of 5FU was close to its half maximal inhibitory concentration (IC50).

Culture media for all groups were aspirated after 24 hours. In order to assess drug toxicity, 100 µL 3-(4, 5-dimethylthiazol-2-yl)-2, 5-diphenyltetrazolium bromide (MTT) with a concentration of 5 mg/ml was then added to each well and incubated for 4 hours at 37°C. After incubation, MTT was pipetted out and 100 µL isopropanol was added and incubated for another 20 minutes until formazan crystals were solved. Absorbance of the solution was measured at 570 nm as a criterion of alive cells^31^ by a fully automatic reading unit of Awareness Technology Stat Fax 2100 Microplate Reader, the US. Cell viability was calculated according to equation 2.^33^


(2) Viability 00=ODSODC×100

Wherein OD_s_ and OD_c_ were the mean of optic density for the samples and negative control, respectively.

### Statistical analysis

The results were statistically analyzed in 95% level of significance using one-way ANOVA and ANCOVA by SPSS 23 software.

## Results and Discussion

### Core-shell nanofibers characterization

#### Morphology

SEM images were used to investigate morphology of various core-shell nanofibers which were electrospun according to Taguchi DEO conditions. Table 2 shows the morphological characterization of core-shell nanofiber mats. One-way ANOVA test results showed that sheath polymer ratio (*P* = 0.039), percentages of Fe_3_O_4_ and 5FU (*P* < 0.001) had a significant effect on viscosity. In other words, the viscosity was increased by increasing CS, 5FU, and decreasing magnetic nanoparticles. Also, increasing CS percentage in sheath polymer ratio remarkably increased conductivity (*P* < 0.001). The number of magnetic nanoparticles (*P* = 0.865) and 5FU (*P* = 0.994) had no effect on conductivity.

**Table 2:** Morphology and tensile properties of core-shell nanofibers mat

**Samples**	**Diameter, nm**	**Sheath viscosity, mPa.s**	**sheath conductivity, µS**	**Tensile properties**		
				**Strength, MPa**	**Strain, %**	**Modulus, MPa**
C1	468±140	951±5	219±4	3.48±0.77	59.56±16.78	35.27±5.99
C2	382±150	622±6	227±3	6.01±2.19	72.61±30.70	32.07±8.53
C3	398±103	805±4	247±5	10.88±1.27	79.01±6.34	78.24±8.57
C4	397±113	1307±7	357±4	6.59±0.37	63.90±4.08	58.45±3.27
C5	347±92	935±5	367±5	9.29±1.81	85.46±7.95	60.26±6.40
C6	290±217	546±7	382±7	7.69±1.06	75.06±3.62	63.29±1.61
C7	311±64	1166±6	638±6	7.05±0.59	72.81±10.69	63.49±8.76
C8	305±66	1317±5	661±4	10.34±1.58	57.36±5.53	76.06±3.60
C9	272±51	831±5	686±5	7.35±1.51	38.87±6.62	87.12±2.16
C10	392±108	767±6	214±5	7.95±1.17	111.78±7.12	36.41±4.48
C11	419±102	990±5	234±6	6.62±0.65	111.04±7.31	36.35±7.17
C12	401±93	621±6	244±4	5.72±1.25	71.53±17.13	42.95±3.79
C13	410±121	1309±4	359±3	6.46±1.75	57.95±2.73	50.52±1.76
C14	403±127	938±3	369±6	8.82±1.31	55.20±7.02	51.57±4.97
C15	399±79	544±7	380±6	6.95±0.24	66.73±5.38	55.67±3.42
C16	349±89	1350±6	643±7	4.98±0.82	78.39±6.37	60.77±2.03
C17	340±93	949±5	669±5	5.52±2.34	63.49±5.62	69.13±5.60
C18	378±140	1014±6	692±6	4.86±0.68	47.15±7.18	71.20±4.07

The results also indicated that feeding ratio of sheath to core and the amount of CS, Fe_3_O_4_, and 5FU had a statistical effect on nanofibers diameters (*P* < 0.001). Increasing feeding ratio caused an increase in diameter. Increasing CS in sheath led to a decrease in diameter due to increasing sheath conductivity in spite of an increase in viscosity.^1,13^ Decreasing Fe_3_O_4_ and increasing 5FU led to an increase in diameter due to an increase in viscosity.^34^ Since the solvent of core and sheath was the same and thus had the same conductivity, nanofibers were thick and diametrically uneven.^35^

#### Tensile properties

Table 2 shows the results for tensile properties. ANOVA test results indicated that feeding ratio had no effect on strength (*P* = 0.061) and strain (*P* = 0.159) while it affected modulus (*P* = 0.033). Increasing feeding ratio decreased modulus as a result of increasing nanofibers diameter.^36^

The results also showed that the amount of CS had no effect on strength (p = 0.316). Nevertheless, changing CS content affected strain and modulus (*P* < 0.001). Increasing CS percentage caused an increase in modulus and a decrease in strain due to presence of hydroxyl groups in CS polymer chain and thus inter-chain hydrogen bonds.^1,36^ Percentage of Fe_3_O_4_ had no effect on strain and stress (*P* > 0.05) unlike modulus (*P* = 0.002). Increasing Fe_3_O_4_ caused viscosity to decrease which led to a decrease in diameter and eventually an increase in modulus. The amount of drug had no effect on tensile properties (*P* > 0.05). Although there are no predefined intervals for tensile properties of nanofibrous drug delivery systems, the results showed that the produced mats were capable of being used as implants.

#### Biodegradability of nanofibrous layer

**Figure 1:**
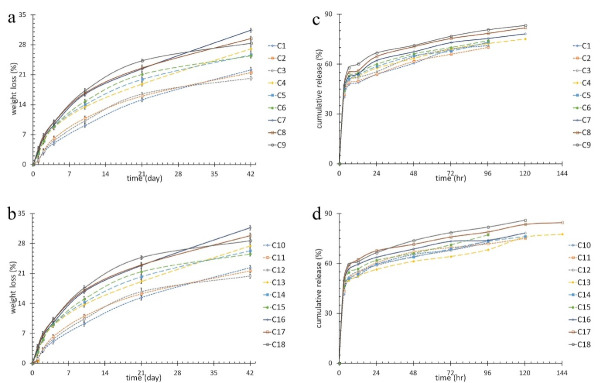
(a) and (b)weight loss percentage of the nanofibrous mats, (c) and (d) cumulative release of 5FU from various nanofibers mat.

The results for weight loss of the mats are shown in Figure 1. Unlike CS content (*P* < 0.001), feeding ratio (*P* = 0.764), Fe_3_O_4_ (*P* = 0.183) and drug (*P* = 0.971) percentage were not statistically effective on biodegradability of nanofibrous layers after 42 days. Increasing CS, as a hydrophilic component, led to an increase in hydrophilicity of the layer which caused more biodegradability.

#### Drug loading efficiency

The ratio of actual amount of drug trapped in the nanofibrous layer to theoretical amount is considered as drug loading efficiency. The results are shown in Table 3. ANOVA test results indicated that unlike CS amount (*P* < 0.001), feeding ratio, drug and magnetic nanoparticle amount (*P* > 0.05) were not statistically effective on drug loading efficiency. Increasing CS amount was directly related to drug loading efficiency. Hydrophilic nature of the CS ^1^ on one hand and presence of amine groups in CS, which can charge them positively,^37^ on the other hand can cause enhanced interaction of CS with negatively charged C = O and C-F bands of 5FU^38^ which are hydrophilic, too.^1^

**Table 3:** Drug loading efficiency and coefficients of the mathematical models

**Samples**	**drug loading efficiency (n=3)**		**Peppas**			**Weibull**		
	**Theoretical**	**Efficiency**	**K**_k_	**n**	**R** ^2^	**a**	**b**	**R** ^2^
C1	3	61.92±1.80	0.34	0.16	0.97	46.7	0.24	0.96
C2	1	64.45±1.37	0.36	0.14	0.98	53.4	0.22	0.96
C3	5	65.91±1.42	0.37	0.14	0.97	38.9	0.23	0.96
C4	5	64.99±1.19	0.39	0.13	0.98	35.7	0.22	0.97
C5	3	67.40±1.08	0.39	0.13	0.99	35.9	0.21	0.98
C6	1	72.19±1.71	0.39	0.14	0.98	30.5	0.23	0.98
C7	1	78.65±1.27	0.39	0.14	0.99	25.0	0.24	0.99
C8	5	81.43±1.60	0.40	0.15	0.99	18.9	0.26	0.98
C9	3	83.56±1.33	0.42	0.14	0.98	15.2	0.26	0.98
C10	1	65.29±1.22	0.36	0.15	0.98	37.3	0.24	0.99
C11	5	66.47±0.94	0.38	0.14	0.99	31.6	0.23	0.99
C12	3	69.36±1.25	0.38	0.14	0.97	34.8	0.22	0.97
C13	5	68.76±1.39	0.39	0.12	0.94	39.8	0.21	0.91
C14	3	73.26±1.11	0.40	0.13	0.98	32.0	0.22	0.97
C15	1	77.21±2.16	0.41	0.13	0.97	24.4	0.22	0.96
C16	3	80.14±1.94	0.43	0.12	0.98	18.9	0.22	0.97
C17	1	83.49±2.08	0.43	0.13	0.98	14.6	0.25	0.98
C18	5	86.14±1.05	0.44	0.14	0.98	12.7	0.26	0.97

#### Drug release

Figure 1 shows the drug release curves of the nanofibrous mats. The release time intervals were 96 to 140 hours. One-way ANOVA test was conducted to study the effects of structural parameters and AMF. The results showed CS content had significant effect on burst release (BR), maximum release time (TR_max_) and its relevant release amount (R_TRmax_) (*P* < 0.001). Increasing CS content increased BR, TR_max_, and R_TRmax_ due to improved interaction of CS and 5FU and therefore an increase in drug trapping. AMF and 5FU amount had no effect on BR, TR_max_, and R_TRmax_ (*P* > 0.05). Feeding ratio had a direct effect on BR, TR_max_, and R_TRmax_ (*P* < 0.05). Increased feeding ratio led to an increase in CS content which ended in better interaction with 5FU. More trapped 5FU led to increased BR, TR_max_, and R_TRmax_. Moreover, increasing feeding ratio caused an increase in nanofiber diameter and hence a decrease in special surface. Magnetic nanoparticles affected TR_max_ (*P* = 0.016), however it had no effect on BR (*P* = 0.117) and R_TRmax_ (*P* = 0.574). Increasing Fe_3_O_4_ decreased viscosity and thus the diameter of nanofibers which led to an increase in special surface. Consequently, larger special surface reduced TR_max_ despite it was not desirable for drug release system. However, increasing Fe_3_O_4_ caused an improvement in hyperthermia functionality. In order to compensate lower TR_max_ and increase release sustainability, core-shell structure was utilized in which a hydrophobic polymer (PCL) was loaded with the drug in the core.

#### Drug release kinetics

It was concluded from the results that Korsmeyer-Peppas, and Weibull models fitted well for modelling release behavior of nanofibers. Table 3 shows the coefficients of the models. According to the derived coefficients for Korsmeyer-Peppas model and considering cylindrical structure for nanofiber, the release kinetic matched Fickian diffusion.^39^

### Modelling core-shell nanofibers structure

**Figure 2:**
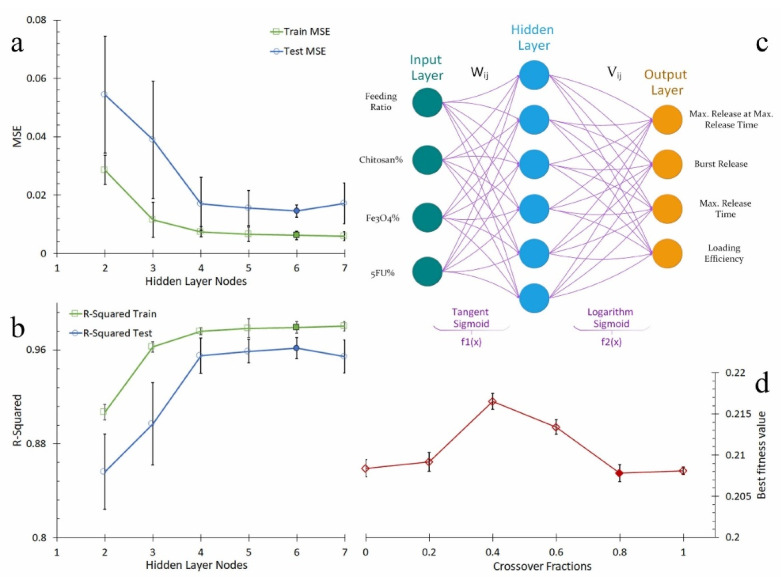
(a) the average of MSE versus nodes number, (b) the average of R^2^ versus nodes number, (c) Optimum neural network structure, and (d) The best fitness value versus crossover fraction.

At first, tangent sigmoid activation functions for all networks with various nodes of 2-7 in hidden layers were defined. Figures 2a and 2b show the average of the MSE and R^2^ for the various nodes respectively. By increasing the number of nodes up to 6, MSE decreased and R^2^ increased for testing and training data. However, for 7 nodes MSE increased and R^2^ decreased. Therefore, 6 nodes in hidden layer were selected as the optimum number of nodes.

Next, in order to determine the best activation functions, Taguchi DOE was performed with 9 permutations. Each permutation was run for 10 times and the average of the MSE and R^2^ were reported. According to Taguchi analysis, tangent sigmoid and logarithm sigmoid functions were considered as input layer and hidden layer output activation functions, respectively. Figure 2c shows optimum neural network structure schematically.

In order to compare Gaussian noise method with cross validation, proper Gaussian coefficients had to be chosen. Thus, the standard deviation with minimum testing error in the range of 0.01 to 0.1 was determined.^25^ Table 4 shows the MSE and R^2^ for cross validation and Gaussian noise methods. Since the latter had smaller MSE and larger R^2^, it was selected as a method to compensate the lack of data. Consequently, 540 data were derived from 54 real data. The derived data along with 18 real data were introduced as training data and 36 remaining real data were considered as testing data. All the data were normalized between 1 and -1 and introduced to ANN in a bipolar form.

**Table 4:** MSE and R^2^ for cross validation and Gaussian noise methods

**Lack of data compensation method**	**MSE**	**R** ^2^
Cross validation (k = 10)	0.0128	0.954
Gaussian noise method (µ = 0, SD = 0.05)	0.0062	0.979

MSE, mean square error.

### Optimizing core-shell nanofiber structure

The fitness function was defined as equation 1 based on ANN outputs values. The optimum amounts for L_e_ and R_TRmax_ were considered 100% and zero for BR. According to the drug release curves, maximum amount of TR_max_ was 144 hours. Thus, the optimum amount was considered 240 hours in fitness function. To determine suitable crossover and mutation fractions, the genetic algorithm was run 10 times for various fractions and the average of the best fitness value was reported. Crossover fraction of 0.8 and mutation fraction of 0.2 were then selected as the best ones according to minimum best fitness value. Figure 2d shows the average of the best fitness value versus crossover fraction.

Afterward, genetic algorithm was run for 20 times. The chromosomes values related to the minimum fitness value were then determined as the optimum number of structural parameters. Feeding ratio of 2, the CS amount of 22% in sheath polymer composition, loaded Fe_3_O_4_ percentage of 5.9%, and loaded 5FU percentage of 4.6% were considered as the optimum amounts of structural parameters and the fitness value for this chromosome structure was 0.2065. Finally, the optimum core-shell nanofiber was produced and investigated. The release behavior of optimum nanofibrous layer was studied in absence of AMF since it had no effect on release behavior.

### Evaluating optimized nanofibers structure

#### Morphological assessment

**Figure 3:**
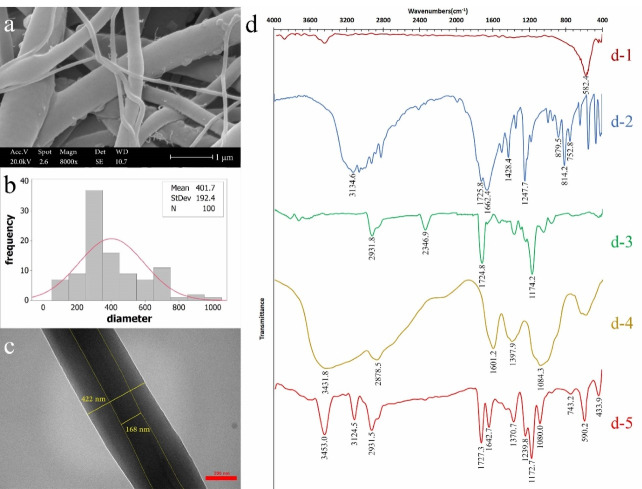
(a) SEM image (scale 2 µm), (b) nanofibers diameter distribution, (c) TEM image (scale 200 nm), and (d)FTIR spectra; (d-1) Fe_3_O_4_, (d-2) 5FU, (d-3) PCL, (d-4) CS, and (d-5) optimum nanofibers containing 5FU and Fe_3_O_4_.

Figures 3a, 3b, and 3c show SEM image, diameter distribution, and TEM image of optimum nanofibers, respectively. The core-sheath structure was approved by TEM image and was continuous. The average diameter of optimum nanofibers was determined 402 ± 192 nm and for the core diameter it was 168 nm in TEM image.

#### FTIR assessment

**Figure 4:**
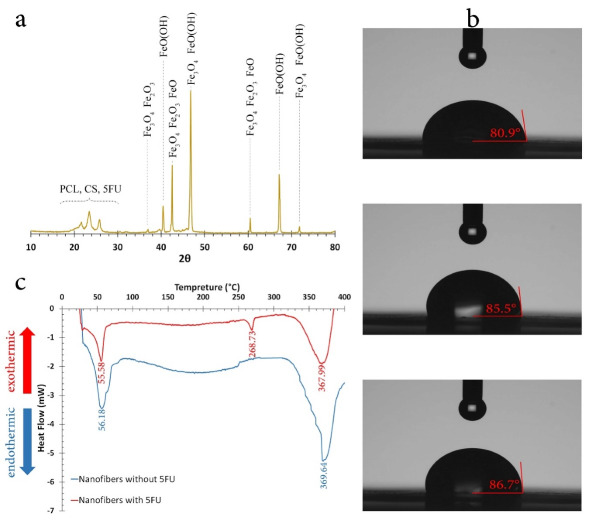
(a) the XRD pattern, (b) Contact angle, and (c) DSC of optimized nanofibers.

**Figure 5:**
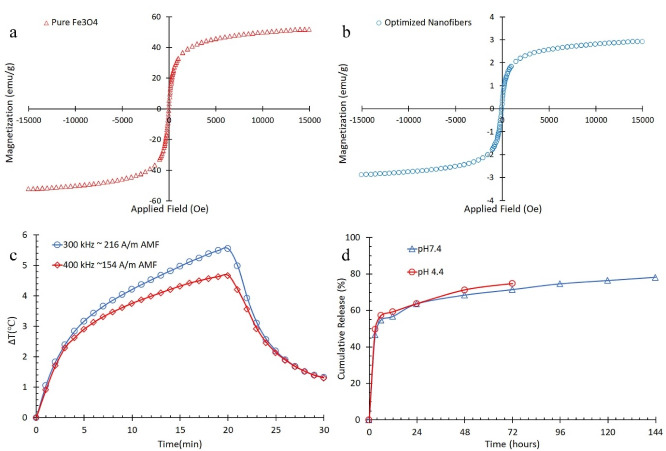
(a) hysteresis M-H curves for pure Fe3O4, (b) hysteresis M-H curves optimized core-shell nanofibers, (c) temperature difference versus magnetic field exposure time, and (d) release behavior of optimized nanofibers.

The chemical structure of optimum nanofibers was confirmed by FTIR. Figure 3d shows the FTIR spectra. A strong peak at 582.4 cm^-1^ was related to Fe-O, which was referred to intrinsic stretching vibration of Fe in tetrahedral site. The peak at 441 cm^-1^ was referred to octahedral-metal stretching of Fe-O. There was no distinguished peak for maghemite phase due to an overlap of maghemite with magnetite phase peaks^13,40^ (Figure 3d-1). The spectrum of 5FU had a distinguished peak at 3134.6 cm^-1^ which was related to N-H stretching vibration. Strong peak at 1725 cm^-1^ referred to C and O double bond and the peak at 1662.4 cm^-1^ related to C = O and C = C. Also, the peaks at 1428.4 cm^-1^ and 1247 cm^-1^ were related to stretching vibration of C-F and C-N, respectively. C-H out of plane deformation vibration was illustrated in peaks at 814.2 and 752.8 cm^-1^ and the peak at 879.5 cm^-1^ related to C-H out of plane bending vibration^41^ (Figure 3d-2). A medium peak at 2931.8 cm^-1^ in PCL spectrum referred to C-H. Strong peaks at 1724.8 and 1174.2cm^-1^ related to C = O and C and O single bond conjugated with C = O in ester functional group in PCL, respectively^42,43^ (Figure 3d-3). The broad and strong peak at 3431.8 cm^-1^ referred to O-H and N-H stretching bond. The peak at 1601.2 cm^-1^ was related to N-H bending vibration. The peak at 1084.3 cm^-1^ referred to C-O and C-N stretch^42,43^ (Figure 3d-4). Figure 3d-5 shows the spectrum for optimum nanofibers containing 5FU and Fe_3_O_4_. The peaks at 3453.0, 1370.7, and 1080.0 cm^-1^ were related to presence of CS. The peaks at 2931.5, 1727.3, and 1172.7cm^-1^ referred to PCL. The peaks related to 5FU were 3124.5, 1624.7, and 1239.8 cm^-1^. The peak at 590.2 cm^-1^ was for the presence of Fe_3_O_4_ and at 433.9 cm^-1^ could be related to the presence of maghemite or wüstite.^13^ The weak peak at 743.2 cm^-1^ may refer to iron (III) oxide-hydroxide.^13^ Fe_3_O_4_ was changed to other iron oxide compositions slightly because of formic acid and Fe_3_O_4_ surface interaction^44^ in preparation of polymer solution. However, there was no interaction between the other components in chemical structure of nanofibers.

#### XRD analysis

The XRD pattern of optimum nanofibers is shown in Figure 4a. As it is shown, various iron oxide phases exist in nanofibers due to formic acid and Fe_3_O_4_ surface interaction which is in agreement with FTIR results. Regarding the similarity of iron oxides XRD patterns, the structure of them could not be ascertained due to peaks overlap.^45^ However, the diffraction peaks of iron oxides can be seen at 36.7°, 40.4°, 42.7°, 46.9°, 60.1°, 66.4° and 71.8°. The peaks at 36.7°, 42.7°, 46.9°, 60.1°, and 71.8° refer to Fe_3_O_4_ assigned to (311), (400), (110), (440), and (620) crystal planes, respectively.^46^ The peaks at 40.4° and 42.7° refer to other iron oxides and the peaks between 20° to 26° determine polymeric structure of CS and PCL.

#### Hydrophilicity

Figure 4b shows the photos of the water droplet on the surface of nanofibers. The average contact angle was 84.4° which showed nanofibers were hydrophilic. Therefore, these nanofibers were more suitable to be used as implants due to better biodegradability as opposed to hydrophobic layers.^47^ Nevertheless, the effect of hydrophilicity in drug release was negligible since drug release followed Fick’s law of diffusion.

#### DSC assessment

Figure 4c shows the DSC curves for optimized nanofibers with and without drug. The endothermic peaks at 56.18 and 369.64°C for nanofibers without drug referred to PCL melting and CS degradation, respectively. For drug loaded nanofibers, the endothermic peaks were at 55.58, 268.73, and 367.99°C for PCL melting, 5FU melting, and CS degradation, respectively. The results indicated that by loading 5FU, PCL melting and CS degradation temperatures decreased insignificantly, which can be due to a decrease in nanofibers crystallinity. Consequently, PCL/CS nanofibers loaded with 5FU and Fe_3_O_4_ were suitable for hyperthermia functional temperature of approximately 45°C.

#### Hyperthermia analysis

VSM test was conducted for pure Fe_3_O_4_ and optimized nanofibers. Figures 5a and 5b show the hysteresis M-H curves for pure Fe_3_O_4_ and optimized nanofibers, respectively. Saturation magnetization for pure Fe_3_O_4_ and optimized nanofibers was calculated by the use of these curves. The actual weighted fraction of magnetic nanoparticles was calculated by equation 3.^13^


(3) $$\phi =\frac{M_{sf}}{M_{sn}}$$

Wherein Ø was the weighted actual magnetic nanoparticles in optimized nanofibers, M_sn_ (52 emu.g^-1^) and M_sf_ (2.9 emu.g^-1^) were saturation magnetization for pure Fe_3_O_4_ and optimized nanofibers, respectively. The actual magnetic nanoparticles per mass unit of nanofibers was 5.6% while nominal amount of loaded Fe_3_O_4_ was 5.9% in optimized nanofibers. This revealed that nanoparticles were not completely loaded and/or they were slightly transformed to other nonmagnetic iron oxides^13^ due to interactions between formic acid and Fe_3_O_4_.^44^ Figure 5c shows temperature increase of optimized nanofibers in the previously mentioned AMFs.

As it is shown, the temperature increased more noticeably after 20 minutes in exposure to the stronger AMF despite lower frequency. The temperature increased about 5.6°C in the AMF of 216 kA.m^-1^, 300 kHz and 4.8°C in the AMF of 154 kA.m^-1^, 400 kHz. Therefore, it can be concluded that the time of AMF exposure can be reduced by increasing the AMF power to reach hyperthermia effect.

#### Release behavior

Since AMF had no effect on release parameters, the release behavior of optimized nanofibers was studied in the absence of AMF in pHs of 4.4 and 7.4 (Figure 5d). TR_max_ was 144 hours in pH of 7.4 while it was 72 hours in pH of 4.4 due to CS sensitivity to acidic ambience.^48^

The results for release parameters of optimized nanofibers, the relevant ANN estimated values, ANN model errors for neutral pH, and the coefficients for Korsmeyer-Peppas and Weibull mathematical models are shown in Table 5.

**Table 5:** Release parameters of optimized nanofibers

**pH**	**R**_TRmax_ **, %**	**TR**_max_ **, h**	**BR, %**	**L**_e_ **, %**	**Mathematical models**					
					**Peppas**			**Weibull**		
					**K**_k_	**n**	**R** ^2^	**a**	**b**	**R** ^2^
7.4	78.22	144	34.41	81.48	0.42	0.13	0.99	20.6	0.23	0.99
4.4	74.87	72	35.74		0.45	0.12	0.97	16.8	0.20	0.97
ANN estimation	86.22	141	36.07	86.22	-	-	-	-	-	-
Error, %	10.65	2.08	4.82	5.82	-	-	-	-	-	-

#### EDX assessment

**Figure 6:**
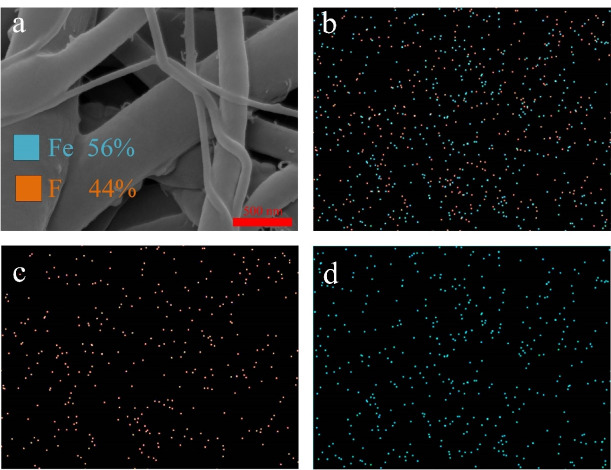
element analysis of the optimized nanofibers; (a) SEM, (b) element mapping of Fe and F, (c) element mapping of F, and (d) element mapping of Fe.

Figure 6 shows the element mapping for the optimized sample. The presence of Fe and F in the mapping confirmed the presence of iron oxides and 5FU. The detected ratio of Fe and F were 0.56 and 0.44, respectively. Since the EDX has the probing depth of 0.5 to 1 µm and gives information about the bulk composition,^49^ it was concluded that 5FU and iron oxide nanoparticles were distributed evenly in the optimized nanofibers.

#### Cell cytotoxicity assay

**Figure 7:**
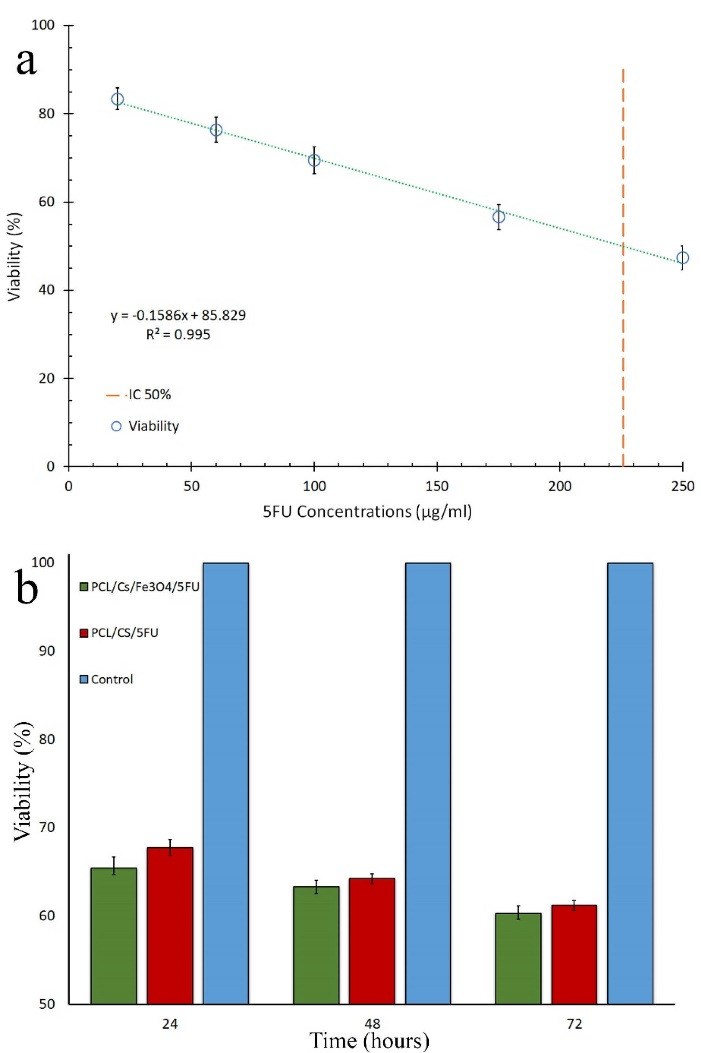
(a) linear relationship between calculated cell viability of HepG2 cultured cells and concentrations of 5FU, and (b) cell cytotoxicity for the optimized samples with and without Fe_3_O_4_ (n = 3).

Figure 7a shows the plot of cytotoxicity against 5FU concentration wherefrom IC50 was calculated as 226 µg/mL. Based on the extraction volume medium and the dimensions of the optimized samples with and without Fe_3_O_4_, the concentrations of 5FU were calculated as 235 and 220 µg/mL, respectively. Cell cytotoxicity for the optimized samples with and without Fe_3_O_4_ after 24, 48, and 72 hours of extraction are shown in Figure 7b. The results showed that cell cytotoxicity of the optimized samples with and without Fe_3_O_4_ after 72 hours were 39.7% and 38.8%, respectively.

ANOVA test results showed that the extraction time had significant effect on cell cytotoxicity of the optimized samples with Fe_3_O_4_ (*P* = 0.001) and without Fe_3_O_4_ (*P* < 0.001). The results showed that the cytotoxicity increased by increasing extraction time. ANCOVA test was applied to show the effect of the presence of Fe_3_O_4_ in optimized samples on cell cytotoxicity during extraction time. The results indicated that cytotoxicity of the samples with and without Fe_3_O_4_ were not statistically different (*P* = 0.097).

## Conclusion

Different structures of core-shell nanofibers based on Taguchi DOE were produced. Morphology, tensile properties, biodegradability, and release behavior of the produced samples were investigated. The results showed that by increasing the amount of CS from 7% to 23% BR, TR_max_, R_TRmax_, and L_e_ increased 4%, 28 minutes, 10%, and 17%, respectively. Increasing Fe_3_O_4_ from 1% to 5% increased TR_max_ for 16min. Changing the amount of Fe_3_O_4_ had no significant effect on BR, R_TRmax_, and L_e_. Apparently, increasing the amount of magnetic nanoparticles enhances hyperthermia functionality.^13^ ANN was then run to estimate release parameters from the structural parameters. Afterward, genetic algorithm fitness function was defined based on ANN. Finally, structural parameters for producing the optimized sample were derived. The release parameters of the produced optimized sample were compared with those of ANN outputs and the errors for BR, TR_max_, R_TRmax_, and L_e_ were approximately 5%, 2%, 10%, and 6% which showed an acceptable compliance. In order to chemically investigate the optimized sample, FTIR, XRD, and EDX were performed. FTIR results showed that there was no interaction between components. However, it was shown that Fe_3_O_4_ was slightly transformed to other iron oxide compositions because of formic acid and Fe_3_O_4_ surface interaction^44^ in preparation of polymer solution. XRD results confirmed other forms of iron oxides. EDX results verified even distribution of Fe and F in the optimized sample. VSM test was conducted to measure the real number of magnetic nanoparticles. The results indicated that the actual magnetic nanoparticles amount was 0.3% less than nominal amount due to Fe_3_O_4_ transformation to other nonmagnetic iron oxides. To investigate hyperthermia effect, optimized sample was exposed to 2 different AMFs. The results suggested that increasing magnetic field intensity by 40% increased the temperature by 16% in a constant AMF exposing time of 20 minutes. Therefore, it was concluded that more powerful AMFs can reach to the hyperthermia temperature in a shorter time. HepG2 cell cytotoxicity was studied for four groups of negative control, positive control, extraction of optimized sample with Fe_3_O_4_, and without Fe_3_O_4_. IC50 was calculated 226 µg/mL. The results showed that cell cytotoxicity of the optimized samples with and without Fe_3_O_4_ after 72 hours were 39.7% and 38.8%, respectively.

Crosslinking CS can help increase TR_max_ and decrease BR which can be done in future works. Moreover, different drugs can be used in core and shell, separately and the release profiles can be studied as well. Another work which can be done in the future is to utilize more Fe_3_O_4_ in more powerful AMFs to achieve more rapid hyperthermia. Finally it seems these nanofibers can be used as post-surgical implants for various cancer treatments such as liver or colorectal cancer, after more comprehensive in vivo studies.

## Ethical Issues

The submission does not report on or involve the use of any animals or human data or tissues, so ethical approval does not applicable.

## Conflict of Interest

There are no conflicts of interest to declare.
